# Sensitivity of cold acclimation to elevated autumn temperature in field-grown *Pinus strobus* seedlings

**DOI:** 10.3389/fpls.2015.00165

**Published:** 2015-03-24

**Authors:** Christine Y. Chang, Faride Unda, Alexandra Zubilewich, Shawn D. Mansfield, Ingo Ensminger

**Affiliations:** ^1^Department of Biology, University of Toronto MississaugaMississauga, ON, Canada; ^2^Graduate Department of Cell and Systems Biology, University of TorontoToronto, ON, Canada; ^3^Department of Wood Science, University of British ColumbiaVancouver, BC, Canada; ^4^Graduate Department of Ecology and Evolutionary Biology, University of TorontoToronto, ON, Canada

**Keywords:** *Pinus strobus*, elevated temperature, freezing tolerance, photosynthesis, photoprotection, carbohydrates, autumn cold acclimation, T-FACE

## Abstract

Climate change will increase autumn air temperature, while photoperiod decrease will remain unaffected. We assessed the effect of increased autumn air temperature on timing and development of cold acclimation and freezing resistance in Eastern white pine (EWP, *Pinus strobus*) under field conditions. For this purpose we simulated projected warmer temperatures for southern Ontario in a Temperature Free-Air-Controlled Enhancement (T-FACE) experiment and exposed EWP seedlings to ambient (Control) or elevated temperature (ET, +1.5°C/+3°C during day/night). Photosynthetic gas exchange, chlorophyll fluorescence, photoprotective pigments, leaf non-structural carbohydrates (NSC), and cold hardiness were assessed over two consecutive autumns. Nighttime temperature below 10°C and photoperiod below 12 h initiated downregulation of assimilation in both treatments. When temperature further decreased to 0°C and photoperiod became shorter than 10 h, downregulation of the light reactions and upregulation of photoprotective mechanisms occurred in both treatments. While ET seedlings did not delay the timing of the downregulation of assimilation, stomatal conductance in ET seedlings was decreased by 20–30% between August and early October. In both treatments leaf NSC composition changed considerably during autumn but differences between Control and ET seedlings were not significant. Similarly, development of freezing resistance was induced by exposure to low temperature during autumn, but the timing was not delayed in ET seedlings compared to Control seedlings. Our results indicate that EWP is most sensitive to temperature changes during October and November when downregulation of photosynthesis, enhancement of photoprotection, synthesis of cold-associated NSCs and development of freezing resistance occur. However, we also conclude that the timing of the development of freezing resistance in EWP seedlings is not affected by moderate temperature increases used in our field experiments.

## Introduction

Global land-surface temperatures are increasing, particularly in northern latitudes and during winter months (Intergovernmental Panel on Climate Change, 2007). Records collected since the mid-twentieth century describe a delay in the onset of dormancy and an increase in growing season length in temperate and boreal forest regions across the northern hemisphere, particularly in North America (Boisvenue and Running, 2006; Piao et al., 2007; McMahon et al., 2010). The environmental signals used by trees to sense seasonality and trigger dormancy and development of cold acclimation are the decrease in temperature and the length of photoperiod during the autumn (Welling et al., 2004). Increasing temperatures, as projected by climate change models, will delay the low temperature signal while photoperiod will remain unaffected. Asynchronous phasing of temperature and photoperiod is expected to impact the onset and development of cold acclimation during autumn. In evergreen conifers from high latitudes, cold acclimation includes the cessation of growth (Rossi et al., 2008), development of bud dormancy (Cooke et al., 2012), changes in chloroplast function and membrane composition (Öquist and Huner, 2003; Ensminger et al., 2006; Crosatti et al., 2012), a transition from dynamic to sustained energy quenching (Demmig-Adams and Adams, 2006), changes in gene expression (Ruelland et al., 2009), accumulation of intracellular metabolite pools (Stitt and Hurry, 2002), and cold hardening (Guy, 1990). A critical factor affecting the impact of future elevated autumn temperature is the importance of photoperiod *vs*. temperature for the induction of phenological events. Early conifer studies suggested seasonal variations in photon flux density (Troeng and Linder, 1982) and the onset of autumn frosts (Bergh et al., 1998) as regulators of autumn phenology. More recent studies identified differences in the sensitivity of various evergreen conifer species to photoperiod and temperature during autumn bud dormancy (Olsen, 2010; Cooke et al., 2012).

Photoperiod induces physiological changes in plants during late summer and early autumn. Decreasing photoperiod results in the depletion of sugars, particularly sucrose, toward the end of the night, as was shown in *Arabidopsis* (Gibon et al., 2009) and *Populus* (Hoffman et al., 2010). The nighttime depletion of sugars acts as a metabolic signal during the following day, inhibiting growth and reducing the rate of starch turnover (Gibon et al., 2009). As temperatures begin to decrease during autumn, low temperature exposure induces the cessation of growth in evergreen conifers by limiting photosynthetic productivity and decreasing the rate of cell differentiation (Rossi et al., 2008). The resulting decrease in carbon sink size affects rates of cellular respiration and induces negative feedback regulation of photosynthetic carbon assimilation (Busch et al., 2007; Bauerle et al., 2012).

Enzymatic reactions of the Calvin-Benson cycle are slowed down under low temperature conditions (Bernacchi et al., 2002). To compensate for the reduced energy sink, evergreen conifers reduce their capacity for harvesting sunlight by adjusting photosynthetic pigment pools, and downregulate the capacity of the light reactions in order to maintain photostasis (Huner et al., 1998; Ensminger et al., 2006; Kurepin et al., 2013). Low temperature also inhibits turnover rates for the reaction center core protein D1 (Schnettger et al., 1994; Öquist et al., 1995; Zarter et al., 2006), thus decreasing the number of functional PSII reaction centers and limiting photochemical energy conversion (Sveshnikov et al., 2006; Zarter et al., 2006). As a result, the plant's capacity to quench absorbed light energy via photochemical energy conversion is greatly diminished (Sveshnikov et al., 2006; Zarter et al., 2006; Busch et al., 2007).

As photochemical efficiency decreases under low temperature conditions, light energy absorbed in excess energy can induce the light harvesting complexes (LHCs) to dissociate from photosynthetic reaction centers (Iwai et al., 2010; Johnson et al., 2011), and trigger the formation of thylakoid protein aggregates (Ottander et al., 1995). Excess light energy can also generate highly reactive chlorophyll and oxygen radicals (Ensminger et al., 2006). Plants increase the production of radical scavengers, such as α-tocopherol, β-carotene, neoxanthin and lutein (Havaux and Kloppstech, 2001; Busch et al., 2007). Xanthophyll pigments also serve a year-round photoprotective function. High light exposure causes the de-epoxidation of violaxanthin, via antheraxanthin, into zeaxanthin. During the warm seasons, this occurs in a dynamic and reversible process known as the xanthophyll cycle, which is involved in energy-dependent nonphotochemical quenching in response to a trans-thylakoid pH gradient created by photosynthetic electron transport (Öquist and Huner, 2003; Ensminger et al., 2006; Sveshnikov et al., 2006; Zarter et al., 2006; Busch et al., 2007). The interaction of zeaxanthin with LHCII, mediated by the PsbS protein (Niyogi et al., 2004), allows excess light energy to be dissipated as heat (Zarter et al., 2006); zeaxanthin also acts as an antioxidant to protect membrane-bound lipids (Johnson et al., 2007). In evergreen conifers, prolonged exposure to cold-induced high light stress arrests the xanthophyll cycle in the zeaxanthin form and induces PsbS accumulation at the LHCII aggregates, allowing absorbed energy to be constantly dissipated in a process known as sustained nonphotochemical quenching (Öquist and Huner, 2003; Demmig-Adams and Adams, 2006; Zarter et al., 2006).

As photosynthesis and growth cease, leaf carbon partitioning is shifted from starch to soluble sugar metabolism, enabling mobilization of carbohydrates from leaves to sink tissues (Guy et al., 1992; Strand et al., 1999; Stitt and Hurry, 2002; Dauwe et al., 2012). In addition to regulating plant metabolism, decreasing photoperiod causes phytochromes to activate a cold response pathway mediated by the CBF transcription factors (Maibam et al., 2013), resulting in enhanced freezing tolerance (Welling et al., 2002, 2004; Li et al., 2003; Lee and Thomashow, 2012). Low temperature induces a stronger cold response via CBF (Cook et al., 2004) and ABA-mediated (Cuevas et al., 2008) pathways, resulting in strengthened cytoskeleton and cell walls, increased membrane lipid fluidity and synthesis of cryo- and osmoprotectants (reviewed in Crosatti et al., 2012), as well as accumulation of soluble sugars including raffinose and sucrose in leaf tissues (Dauwe et al., 2012). High levels of sucrose (Tabaei-Aghdaei et al., 2003) and raffinose (Pennycooke et al., 2003) are correlated with increased freezing tolerance.

Several studies have investigated the effect of elevated temperature on plants and growing season length. Most studies have focused on the effects of spring warming (Hänninen and Tanino, 2011). Studies assessing the response of evergreen conifers to elevated autumn temperature have largely been conducted using climate chambers (e.g., Busch et al., 2007), mesocosms (e.g., Tingey et al., 2007) or open-top chambers (e.g., Murray et al., 1994; Wang et al., 1995; Repo et al., 1996; Guak et al., 1998). However, results obtained from chamber experiments often cannot be directly extrapolated to the field (Aronson and McNulty, 2009). Temperature free-air-controlled enhancement (T-FACE) experiments provide an attractive alternative to chamber systems because they do not affect solar radiation, precipitation, soil or wind (Kimball et al., 2008; Aronson and McNulty, 2009). Previous T-FACE experiments have focused on herbaceous species, such as wheat (de Boeck et al., 2012), alfalfa (Kimball et al., 2008), rice (Mohammed and Tarpley, 2009), and prairie grasses (Luo et al., 2001; Kimball et al., 2008). Studies using T-FACE experiments involving evergreen conifer seedlings have been rare and focused on the effect of elevated temperature on productivity during the growing season (e.g., Zhao and Liu, 2009).

The aim of this study was to characterize autumn cold acclimation in the evergreen conifer *Pinus strobus* under field conditions and to assess the effect of elevated autumn temperature at the beginning of the cold hardening process and the subsequent development of cold hardiness. We hypothesized that elevated temperature (i) delays the downregulation of photosynthesis, (ii) delays the transition from dynamic to winter sustained non-photochemical quenching, (iii) delays changes in non-structural leaf carbohydrates including starch and low temperature-associated soluble sugars, and (iv) impairs the development of freezing tolerance. A T-FACE system was used to increase temperature by 1.5°C during the day and 3°C during the night, in accordance with 35-year temperature projections for the Canadian provinces of Ontario and Québec (Price et al., 2011).

## Materials and methods

### Study site and plant material

The experiment was conducted at the Koffler Scientific Reserve of the University of Toronto located near King City, Ontario (44°050′N, 79°483′W). A Temperature Free-Air-Controlled Enhancement (T-FACE) system was set up according to Kimball et al. (2008), consisting of 10 experimental plots, each with a diameter of 3 m. Ambient canopy temperature (AT) was recorded using infrared sensors (Model IRT-P5, Apogee Instruments, Logan, UT, USA) in five unheated control plots. For the elevated temperature (ET) treatment, five plots were arranged with six 1000 W infrared heaters (Mor Electric Heating Association, Comstock Park, MI, USA) per plot in a hexagonal array, where leaf temperature was raised by +1.5°C during the day and +3°C during the night, according to Kimball et al. (2008). Ambient air and canopy temperatures were recorded using a CR1000 datalogger (Campbell Scientific Inc., Edmonton, AB, Canada). Precipitation data were obtained from the Buttonville Airport weather station in Newmarket, ON (Environment Canada, 2014), located 25 km from the field site.

**Figure 1:**
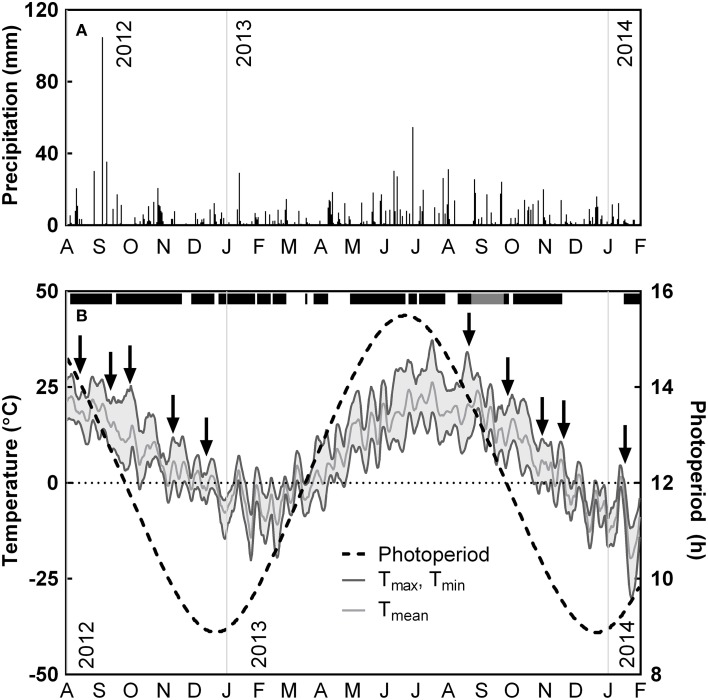
**Seasonal variations in precipitation, daylength and temperature from August 1, 2012 to January 31, 2014 at Koffler Scientific Reserve in Ontario, Canada**. **(A)** Daily precipitation and **(B)** 5-day running averages of max (upper dark gray line), mean (light gray line), and minimum ambient air temperature (lower dark gray line), as well as photoperiod (dotted black line). Arrows indicate measuring dates. Black bar above temperature data indicates periods where heaters were operating; gray bar indicates period where heating was on, but data was lost due to a logger malfunction.

Plots were excavated 30 cm deep, filled with a mixture composed of one-third peat, one-third sand and one-third local soil, and tilled prior to planting. Three-year-old (3 + 0) bare-rooted *Pinus strobus* seedlings were obtained from a local seed orchard (seed zone 37, Somerville Nurseries, Everett, ON, Canada). In early May 2012, 90 seedlings were planted per plot. Gas exchange and fluorescence measurements commenced in mid-August 2012 after seedlings had established, and continued until December 2012. During 2013, measurements were expanded to assess water potential, soil moisture, and freezing tolerance. Measurements in 2013 were taken monthly from August 2013 until November, with final measurements taken in January 2014 (Figure 1). At each time point, three seedlings were randomly selected for measurement from each of five replicate plots per treatment. Soil moisture was measured using a HydroSense ™ soil water content sensor (Campbell Scientific Inc., Edmonton, AB, Canada). Soil moisture, measured as percent volumetric water content, was assessed at a depth of 15, 10 cm from the base of each measured seedling, three times per seedling. Deep frozen soil and ice packs prevented measurements of soil moisture in January 2014. Air humidity and temperature sensors (Hoskin Scientific Limited, Burlington, ON, Canada) were installed in May 2014, in order to assess differences in vapor pressure deficit (VPD), or the difference between actual and saturated air moisture, between heated and unheated plots during July 2014.

Mature current-year needles were collected from measured trees immediately following measurements, flash-frozen in liquid nitrogen, and stored at −80°C until analysis.

### Photosynthetic gas exchange and chlorophyll fluorescence

Gas exchange and chlorophyll fluorescence measurements were performed simultaneously using a portable photosynthesis system (LI-6400 XT; Li-Cor Biosciences, Lincoln, NE, USA) with attached leaf chamber fluorometer (6400-40). Topmost, south-facing needles of the primary shoot were arranged in a flat single-needle layer and placed into the cuvette. The cuvette was set to maintain a level of 400 ppm CO_2_ and ambient temperature, which was selected based on the predicted daily average (Table 1).

**Table 1:** **Cuvette air temperature measured during each measurement campaign, using the LI- 6400 XT gas exchange system**.

	**Cuvette air temperature (°C)**	
**Month**	**2012**	**2013**
August	25.4±0.1	26.2±0.3
September	22.3±0.4	24.7±0.2
October	17.2±0.1	6.3±0.4
November	7.2±0.1	6.5±0.7
December	0.9±0.1	n/a
January	n/a	−0.1±0.1

*Values represent the average of 30 measurements (15 from ambient plots, 15 from elevated temperature plots) ± S.E*.

Dark-adapted minimum PSII fluorescence (*F_o_*), and dark-adapted maximum PSII fluorescence (*F_m_*) were determined after 40 min of dark adaptation. Subsequently, plants were exposed to 1200 μmol quanta m^−2^ s^−1^ for 7–12 min to obtain measurements of steady-state photosynthesis; this light intensity represents one that is typically observed in boreal environments on clear and sunny days, even during early winter or early spring (Ensminger et al., 2004). Measured parameters included photosynthetic CO_2_ assimilation (*A*), stomatal conductance (*g_s_*), evapotranspiration (*E*), light-adapted minimum PSII fluorescence (*F′_o_*), light-adapted maximum fluorescence (*F′_m_*), and transient fluorescence (*F_t_*), which were used to calculate gas exchange and fluorescence parameters (Table 2).

**Table 2:** **Equations for gas exchange and fluorescence parameters**.

**Parameter**		**Equation**	**References**
IWUE	Intrinsic water use efficiency	$\frac{A}{g_{s}}$	Silva and Horwath, 2013
*F_v_*/*F_m_*	Maximum quantum efficiency of PSII	$\frac{F_{m}-F_{o}}{F_{m}}$	Genty et al., 1989
1-qP	Excitation pressure at PSII	$1-\frac{{F^{\prime }}_{m}-F_{t}}{{F^{\prime }}_{m}-{F^{\prime }}_{o}}$	Maxwell and Johnson, 2000
Φ_PSII_	Effective quantum yield of PSII	$1-\frac{F_{t}}{{F^{\prime }}_{m}}$	Genty et al., 1989
NPQ	Total nonphotochemical quenching	$\frac{F_{m}rec}{F_{m^{\prime }}}-1$	Bilger and Björkman, 1990; Ensminger et al., 2004; Porcar-Castell, 2011
NPQ_S_	Sustained nonphotochemical quenching	$\frac{F_{m}rec}{F_{m}}-1$	Maxwell and Johnson, 2000; Ensminger et al., 2004; Porcar-Castell, 2011

The seasonal depression of *F*m due to low temperature does not allow for recovery of the maximum fluorescence signal in the dark, and thus limits its use for the calculation of the fluorescence parameter NPQ (Demmig-Adams and Adams, 2006). A good estimation of NPQ requires a dark-adapted control value of *F_m_* that is measured when the photosynthetic apparatus is in a fully relaxed state. During winter, when *F_m_* is depressed and does not relax rapidly in the dark, NPQ will be underestimated (Demmig-Adams et al., 2012). The non-photochemical quenching parameter, NPQ, was therefore calculated as shown in Table 2. The fully recovered maximum fluorescence (*F_m_rec*) was estimated as *F_o_* * 5, according to Schreiber et al. (1995) and Ensminger et al. (2004). This estimation is based on two assumptions: firstly, the ratio of fully recovered *F_m_*/*F_o_* is equal to 5, which has been demonstrated in multiple plant species (Björkman and Demmig, 1987), including conifers (Adams and Demmig-Adams, 1994); and secondly, unlike *F_m_*, *F_o_* shows little seasonal variation (Ottander et al., 1995). However, this approach might occasionally underestimate NPQ when *F_o_* is strongly decreased.

Each measurement took approximately 15 min and were taken from 2 h after dawn until 2 h prior to sunset. Measurement campaigns occurred over 2–3 consecutive days. Measurement order was randomized at individual, plot and treatment levels during each campaign in order to minimize confounding diurnal or daily effects.

All measurements were performed on attached needles. Following measurement, needles in the cuvette were harvested to estimate the light-exposed needle surface area using a scanner and the WinSeedle software package (Regent Instruments Inc., Québec, QC, Canada).

### Water potential

Water potential measurements were performed from August to November, 2013. Pre-dawn and midday (noon) water potential (Ψ_w_) were assessed on individual current-year needles using a Model 1505D Pressure Chamber Instrument (PMS Instrument Company, Albany, OR, USA). During each campaign, measurements were taken from three needles per seedling on three seedlings per plot, five plots per treatment. During November and January water potential was not assessed because the system did not operate at sub-freezing temperatures.

### Analysis of photosynthetic pigments

50–60 mg homogenized frozen needle tissue was extracted in 2 mL methanol buffered with 2% 0.5 M ammonium acetate according to Junker et al. unpublished. Samples were filtered using a 0.45 μm nylon filter prior to HPLC analysis. Chlorophylls and carotenoids were separated on a reverse-phase C30 column (YMC Carotenoid; Chromatographic Specialties Inc., Brockville, ON, Canada). Pigment extracts were analyzed with an Infinity 1200 series high performance liquid chromatography (HPLC) system equipped with a UV-diode array detector (Agilent Technologies, Santa Clara, CA, USA). De-epoxidation state (DEPS) was calculated as (0.5*A*+*Z*)/(*V+A+Z*) where *V* is violaxanthin, *A* is antheraxanthin, and *Z* is zeaxanthin. Total chlorophylls and α-tocopherol were expressed on a per freshweight basis, as the water content of white pine needles fluctuates less than 10% year-round (Verhoeven et al., 2009).

### Analysis of non-structural carbohydrates

30-40 mg homogenized and lyophilized needle tissue from samples collected in August, October and December of 2012 were extracted in methanol:chloroform:water (12:5:3) according to Park et al. (2009), with the addition of 250 μg galactitol as an internal standard. 2 mL of the soluble sugar extract was vacuum centrifuged and resuspended in 1 mL of nanopure water. The resuspended extract was filtered using a 0.45 μm nylon filter and analyzed using a DX-600 anion-exchange HPLC (Dionex, Sunnyvale, CA, USA) equipped with a Hi-Plex Ca column (Agilent Technologies, Santa Clara, CA, USA) and electrochemical pulse amperometric detector (EC-PAD). Sucrose, fructose, glucose and pinitol were eluted with water at a flow rate of 0.170 mL/min with a column temperature of 70°C. Post-column detection was performed using NaOH at a rate of 100 mM/min. Raffinose was eluted using a Carbo-Pac PA1 column (Dionex, Sunnyvale, CA, USA) with 150 mM NaOH (isocratic) at a flow rate of 1 mL/min with post-column detection using NaOH at a rate of 100 mM/min.

Starch was determined from the residual tissue pellet from the soluble sugar extraction. The pellet was dried overnight at 55°C. 25–50 mg of the dried pellet were resuspended in 5 mL of 4% H_2_SO_4_, vortexed and autoclaved for 3.5 min. After cooling to room temperature, the extract was spun at 500 rpm for 5 min and the supernatant collected. The supernatant was filtered using a 0.45 μm nylon filter and analyzed using a DX-600 anion-exchange HPLC (Dionex, Sunnyvale, CA, USA) equipped with a Carbo-Pac PA1 column (Dionex, Sunnyvale, CA, USA) and EC-PAD. Glucose was eluted with water at a flow rate of 1 mL/min with a column temperature of 30°C. Post-column detection was performed using NaOH at a rate of 100 mM/min.

### Freezing tests

Chlorophyll fluorescence was used to assess freezing tolerance in August, September, October, November of 2013 and January 2014, using a modified protocol based on Sutinen et al. (1992). Current-year shoots were dark-adapted for 40 min. Each shoot was excised and *F_v_*/*F_m_* was measured. The shoots were then individually wrapped in moist paper towel and aluminum foil and sealed in a plastic bag prior to transport on ice back to the laboratory.

Shoots were exposed to freezing temperatures using a Thermotron SM-16-8200 environmental test chamber (Thermotron Industries, Holland, MI, USA). The maximum cooling rate was 2.5°C h^−1^, with the 0 to −1°C interval achieved over 1 h. Since freezing resistance varies over the course of the year, preliminary freezing tests were performed throughout the year to identify a range of freezing temperatures suitable to induce freezing damage in white pine seedlings. Target freezing temperatures were then adjusted during each month of the experiment in order to account for the expected change in freezing tolerance, with the aim of selecting a range of freezing temperatures that bracketed the temperature at which 50% of the seedlings were damaged by freezing (LT_50_). One shoot per tree per freezing temperature was held at the desired temperature for 6–8 h and subsequently thawed in a stepwise manner to room temperature: shoots exposed to ≤ −30°C were kept at −20°C for 24 h, transferred to 4°C for 24 h, and then transferred to room temperature for 24 h recovery. Shoots exposed to ≥ −20°C were transferred directly to 4°C for 24 h and then to room temperature for recovery (Sutinen et al., 1992). Following the 24 h recovery period, shoots were unwrapped and exposed to 1 h light exposure at 800 μmol quanta m^−2^ s^−1^ in order to stimulate PSII, then dark-adapted for 40 min (Burr et al., 2001). *F_v_*/*F_m_* was then assessed. Since we used chlorophyll fluorescence to evaluate freezing injury at PSII, we defined LT_50_ as the temperature required to reduce maximum *F_v_*/*F_m_* by 50%. Maximum *F_v_*/*F_m_* was assessed by subjecting non-frozen shoots to the same protocol of 24 h recovery period, 1 h of light exposure, 40 min of dark adaptation and measurement. LT_50_ values were calculated by fitting *F_v_*/*F_m_* values measured from freezing-recovered shoots using a modified Richards curve model (Fircks and Verwijst, 1993):

$$f\text{​​}\left(x\right)=\frac{K}{1+e^{-B(x-M)}}$$

where K represents the upper asymptote, or pre-freezing *F_v_*/*F_m_*; B represents the maximum slope at LT_50_ and M represents LT_50_. Data was tested for normality using the D'Agostino-Pearson omnibus normality test. The curve for each treatment (elevated *vs*. ambient temperature) was fitted using the least squares method. LT_50_ values were compared between treatments using an extra sum-of-squares *F* test with a *P*-value cutoff of 0.05. Analysis was performed using Graphpad Prism v6.04 (Graphpad Software, Inc., La Jolla, CA, USA).

### Statistical analyses

Two-Way ANCOVA was used to assess the effect of the elevated temperature treatment and time on gas exchange, fluorescence and photosynthetic pigments, while accounting for the effect of seasonal variation introduced by photoperiod and daily temperature. The ANCOVA model used treatment and day of year as categorical fixed factors, photoperiod and minimum daily temperature as continuous numeric covariates, and plot and year as random factors, using the *lme4* package in R v3.1.1 (http://www.r-project.org/). Multiple comparisons were used to contrast treatment within each time point, and were performed using the *multcomp* package in R v3.1.1. *P*-values for multiple comparisons were adjusted using Bonferroni correction.

Starch and soluble sugars were analyzed using Two-Way ANOVA to identify treatment, time and interaction effects. Tukey's HSD *post-hoc* test was used to identify significantly different groups. The statistical analyses for sugars were performed using Graphpad Prism v6.04.1.

Treatment responses of *A, F_v_*/*F_m_*, and NPQ_*S*_ from both years were pooled, independently plotted against minimum daily temperature and photoperiod, and fitted using the least squares method with a 4-parametric sigmoidal curve function:

$$f\text{​​}\left(x\right)=A+\frac{K-A}{1+e^{-B(x-M)}}$$

where K represents the maximal parameter value; A represents the minimal parameter value; B represents the maximum slope and M represents the midpoint of the curve at which estimated values represent 50% of the maximum value of the parameter. R^2^ and 95% confidence intervals were calculated. Midpoints were compared between treatments using a sum-of-squares F test. Modeling and analyses of the sigmoid curves were performed using Graphpad Prism v6.04.1.

## Results

### Seasonal weather patterns

The field site experienced higher amounts of precipitation during the growing season and lower amounts during winter (Figure 1A). 2012 was characterized by a warm early autumn, with daily maximum temperatures remaining above 20°C until the first week of October (Figure 1B). In contrast, during 2013, daily maximum temperatures began to decline below 20°C by the first week of September. Daily mean temperatures remained above 0°C until November in both years. The first night frost was recorded on October 8 in 2012 and on October 27 in 2013. During October 2012, the temperature dropped rapidly until mid-November and remained between a daily minimum of −5°C and a daily maximum of 10°C until mid-December. In contrast, nighttime temperatures during October and early November 2013 were mild, with minimum temperatures only reaching −2°C and daily maximums above 10°C. Minimum temperatures did not reach −20°C during the winter of 2012 until January 1, while minimum temperature reached −20°C on December 12 in 2013 (Figure 1B).

The variation in weather conditions affected temperature and precipitation during measurement campaigns. Measurements taken during August 2012 occurred after several rainy days, whereas measurements taken in August 2013 were taken after 10 days without rainfall (Figure 1A), resulting in decreased soil water content (**Figure 4A**). In 2012, we recorded a daily mean temperatures of 22°C during our measurements in August, 15°C in September and 15°C in October. In contrast, during 2013 we recorded daily mean temperatures of 20°C during our measurements in August, 7°C in September and 4°C in October (Figure 1B).

### Photosynthetic gas exchange

**Figure 2:**
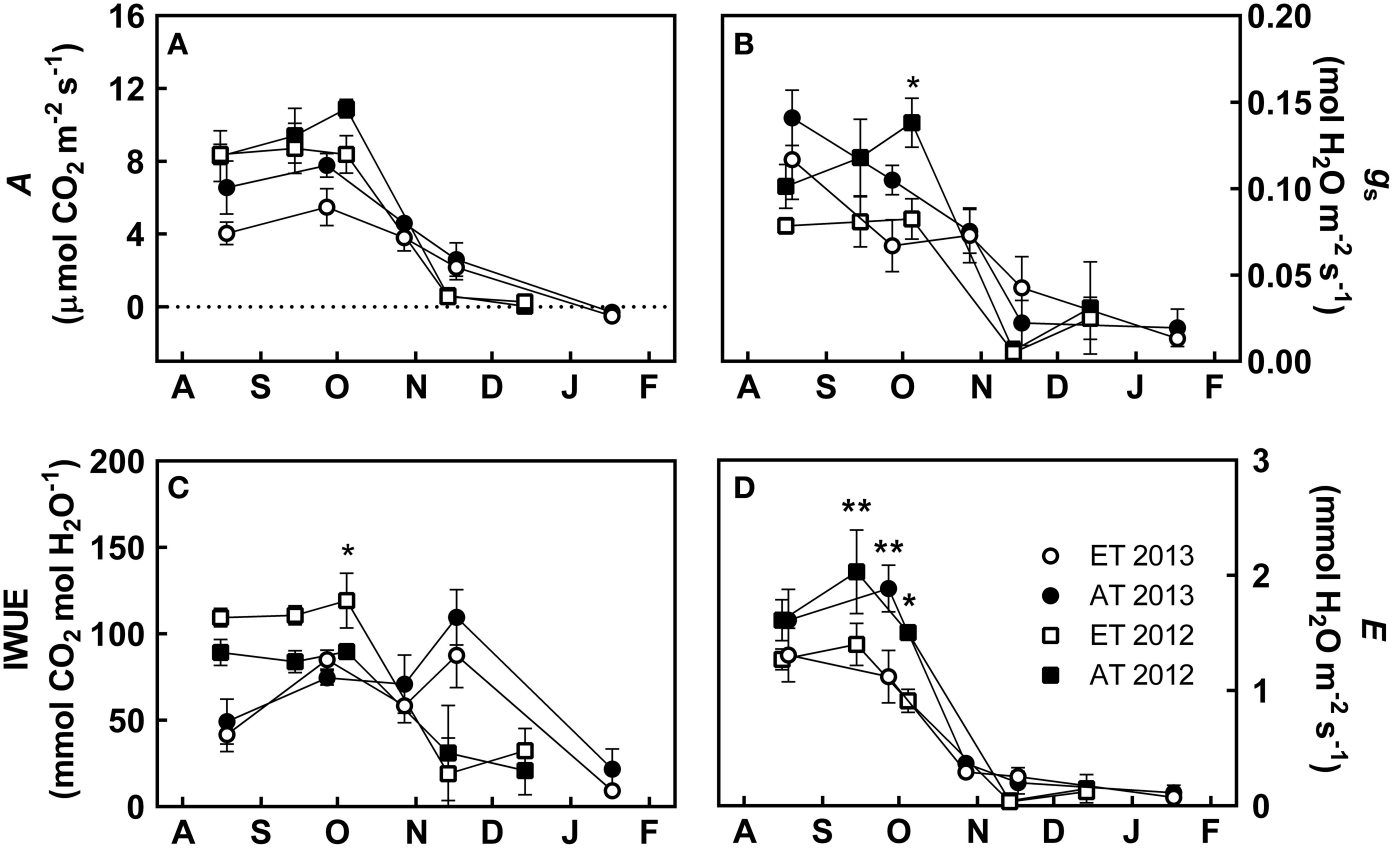
**Effect of elevated temperature on photosynthetic gas exchange in field-grown white pine seedlings during autumn**. **(A)** Photosynthetic carbon assimilation (*A*); **(B)** stomatal conductance (*g_s_*); **(C)** intrinsic water use efficiency (IWUE); **(D)** evapotranspiration (*E*). AT and ET, seedlings grown at ambient and elevated temperature, respectively. Each data point represents the average of 5 plots, ± S.E. Asterisks represent significant treatment effect at a single measuring date (^**^P < 0.01, ^*^P < 0.05).

Photosynthetic carbon assimilation (*A*) remained unchanged from August to the beginning of October, was downregulated during October and November, and eventually ceased in December and January (Figure 2A). This trend was also observed for stomatal conductance (*g_s_*), intrinsic water use efficiency (IWUE) and evapotranspiration (*E*) (Figures 2B–D). However, we also observed differences between years, e.g. during August and September 2013, when we measured lower rates of *A*, *g_s_*, and IWUE (Figures 2A–C) compared to 2012. In 2012, photosynthetic gas exchange was fully downregulated by mid-November, while photosynthetic activity was still detectable in November 2013 (Figures 2A–C). Treatment had a significant effect on *g_s_*, IWUE, and *E*; the interaction of treatment and time significantly affected *g_s_*, IWUE and *E* (Table 3).

**Table 3:** **Summary of Two-Way ANCOVA analysis showing the effects of treatment and time (day of year) on gas exchange, chlorophyll fluorescence, and photosynthetic pigments**.

	**Treatment**			**Time**		**Treatment × Time**	
	**Variable**	***F***	***P***	***F***	***P***	***F***	***P***
Gas exchange	A	2.420	0.121	0.765	0.385	0.809	0.369
	g_s_	**7.361**	**0.007**	0.292	0.589	**4.911**	**0.028**
	IWUE	**4.687**	**0.032**	0.010	0.919	**4.684**	**0.032**
	E	**12.254**	**0.001**	0.048	0.827	**8.302**	**0.004**
Chlorophyll fluorescence	F_*v*_/F_*m*_	1.709	0.192	**201.118**	**<0.001**	1.368	0.243
	1-qP	0.403	0.526	**9.681**	**0.002**	1.357	0.245
	Φ_PSII_	3.572	0.060	1.669	0.198	1.802	0.181
	NPQ	**5.617**	**0.019**	1.343	0.248	2.637	0.106
	NPQ_*S*_	1.476	0.225	**200.188**	**<0.001**	1.073	0.301
Photosynthetic pigments	Total Chl	0.094	0.759	0.532	0.466	0.0516	0.8205
	Chl a/b	**4.012**	**0.046**	**10.905**	**0.001**	3.1231	0.0783
	Car/Chl	0.060	0.808	0.795	0.374	0.4183	0.5183
	α-Car/Chl	1.593	0.208	**3.954**	**0.048**	3.0065	0.0841
	β-Car/Chl	1.137	0.287	**28.745**	**<0.001**	1.8663	0.1730
	V+A+Z/Chl	0.006	0.938	1.233	0.269	0.1311	0.7176
	DEPS	1.298	0.256	**106.087**	**<0.001**	1.2300	0.2683
	Lut/Chl	0.135	0.714	0.031	0.862	0.7057	0.4016
	Neo/Chl	0.013	0.910	**11.269**	**0.001**	0.0251	0.8742
	α-Toc	0.059	0.809	0.908	0.341	0.1099	0.7405

Variables were estimated as Variable ~ Treatment * Time + Photoperiod + Temperature + (1|Plot) + (1|Year). Treatment and time were included as categorical fixed factors. Photoperiod and daily temperature were included as continuous numeric covariates. Plot and year were included as random factors. P-values in bold indicate statistical significance (α = 0.05).

From August to early October, seedlings in heated plots that experienced elevated temperature (ET) exhibited lower *A* in comparison to seedlings in unheated control treatment (Control) that were exposed to ambient temperature (Figure 2A). However, the timing of the autumn downregulation of photosynthesis was not affected by the elevated temperature treatment, as *A* began to decrease by the end of October during both years, irrespective of treatment (Figure 2A). Between August and October of both years, *g_s_* was decreased by 20–30% in ET seedlings compared to Control seedlings. Control seedlings maintained *g_s_* between 0.10 and 0.15 mol H_2_O m^−2^ s^−1^, while the elevated temperature treatment exhibited values between 0.06 and 0.12 mol H_2_O m^−2^ s^−1^ (Figure 2B). *g_s_* was significantly reduced in ET seedlings in heated plots in October 2012 (*P* = 0.041, Figure 2B). In 2012, IWUE was increased by about 15–20% in seedlings in the heated plots compared to Control seedlings, and was significantly enhanced in October (*P* = 0.036, Figure 2C). However, in 2013, IWUE was not significantly affected in seedlings in the heated plots (Figure 2C). *E* was reduced by 20–30% in ET seedlings in heated plots compared to Control seedlings during both years (Figure 2D), particularly during September 2012 (*P* = 0.009), October 2012 (*P* = 0.048), and September 2013 (*P* = 0.003).

### Chlorophyll fluorescence

**Figure 3:**
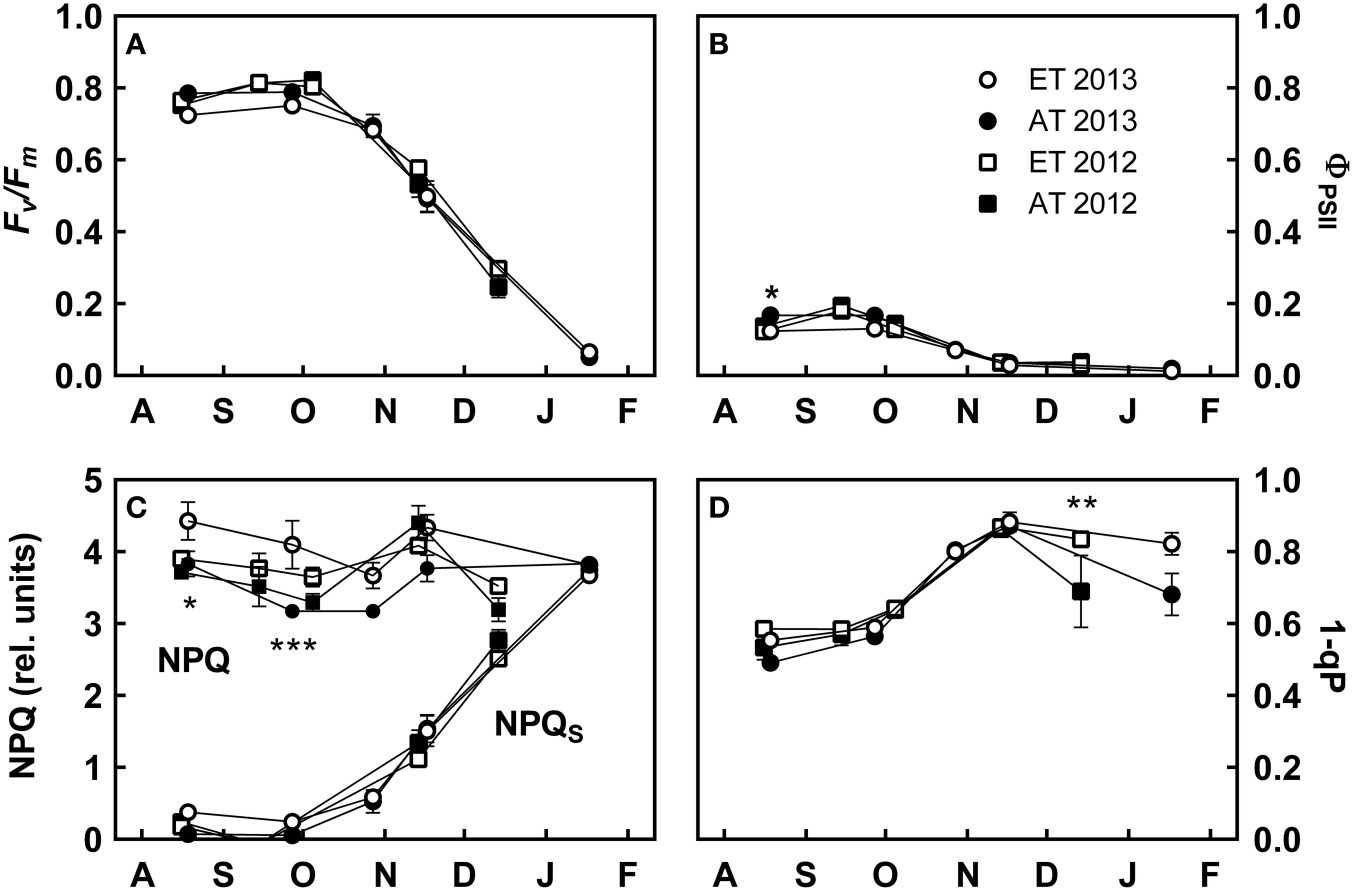
**Effect of elevated temperature on chlorophyll fluorescence in field-grown white pine seedlings during autumn**. **(A)** Maximum quantum yield of PSII (*F_v_*/*F_m_*); **(B)** effective quantum yield of PSII (Φ_PSII_); **(C)** rate constant of total nonphotochemical quenching (NPQ) and sustained nonphotochemical quenching (NPQ*_S_*); **(D)** excitation pressure at PSII (1-qP). AT and ET, seedlings grown at ambient and elevated temperature, respectively. Each data point represents the average of 5 plots, ± S.E. Asterisks represent significant treatment effect at a single measuring date (^***^P < 0.001, ^*^P < 0.05).

The maximum quantum efficiency of PSII (*F_v_*/*F_m_*) was approximately 0.75–0.80 from August to early October, and continuously decreased from late October through January (Figure 3A). The effective quantum yield of PSII (Φ_PSII_) was downregulated during the autumn transition and reached minimum values toward the end of November (Figure 3B). Non-photochemical quenching (NPQ) was high during both years; sustained NPQ (NPQ*_S_*) began to develop during late October and comprised nearly 100% of nonphotochemical processes by January (Figure 3C). Excitation pressure (1-qP) increased during October and reached maximum levels in November before relaxing again in December (Figure 3D). In contrast to the substantial interannual variation observed in photosynthetic gas exchange, we did not observe interannual variations in most fluorescence parameters (Figure 3).

Φ_PSII_ was significantly lower in August 2013 in the elevated temperature treatment (*P* = 0.015, Figure 3B). NPQ was significantly higher in August (*P* = 0.014) and September 2013 (*P* < 0.001) in the elevated temperature treatment, but was not significantly different during 2012; NPQ*_S_* was not significantly different between treatments during either year, although ET seedlings in the heated plots exhibited decreased NPQ*_S_* during November and December (Figure 3C). 1-qP relaxed considerably from November to January under ambient temperature conditions, but did not when exposed to elevated temperature (*P* = 0.002, Figure 3D). Treatment had a significant effect on NPQ, whereas time had a significant effect on NPQ*_S_*, *F_v_*/*F_m_*, and 1-qP (Table 3).

### Water potential and vapor pressure deficit

**Figure 4:**
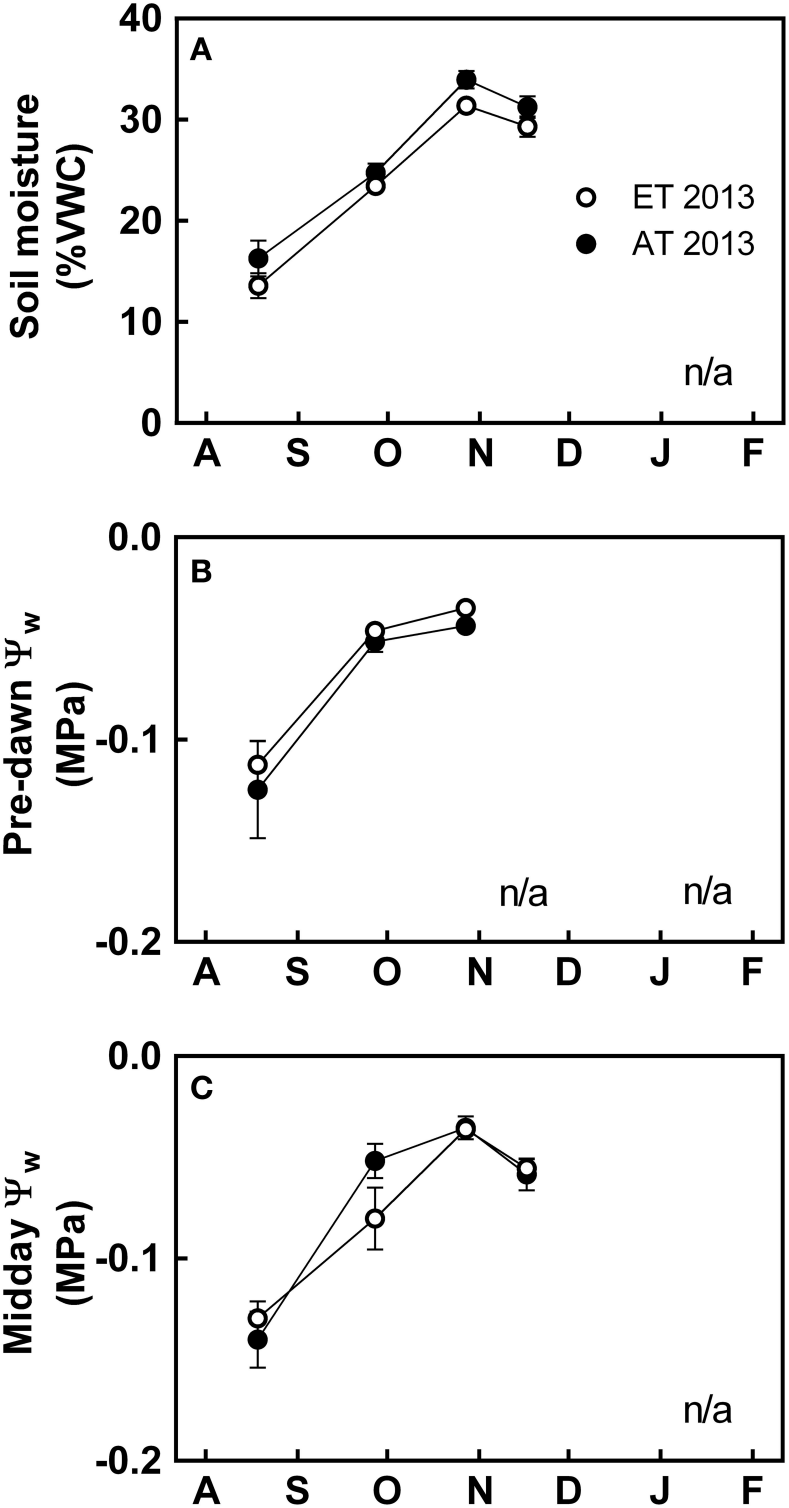
**Effect of elevated temperature on soil water availability and osmotic stress in field-grown white pine seedlings during autumn**. **(A)** Soil moisture content, expressed in percent volumetric water content (% VWC); **(B)** pre-dawn water potential (Ψ_w_); and **(C)** midday Ψ_w_ measured during 2013. AT and ET, seedlings grown at ambient and elevated temperature, respectively. n/a indicates points where water potential was not assessed because the equipment did not operate at sub-freezing temperatures in the field. Each data point represents the average of 5 plots, ± S.E.

In 2013, soil moisture was lowest during August (Figure 4A). There was a consistent but non-significant reduction in soil moisture in the heated plots during the growing season (Figure 4A). Leaf water potential (Ψ_w_) was generally high, with values consistently higher than −0.2 MPa (Figures 4B,C). There was no significant difference in Ψ_w_ during pre-dawn or midday (Figures 4B,C).

The extent of vapor pressure deficit (VPD) imposed by our heating treatment was assessed from July 18 to August 12, 2014 (Figure S1). The difference in VPD between heated and control plots varied depending on air temperature (Figures S1A,B). During the night, an increment of +3°C induced a 20% increase in VPD (0.351 ± 0.089 kPa) in the heated plots, while during the day an increment of +1.5°C induced a 6% increase in VPD (0.179 ± 0.089 kPa) in the heated plots (Figure S1).

### Photosynthetic pigments

**Figure 5:**
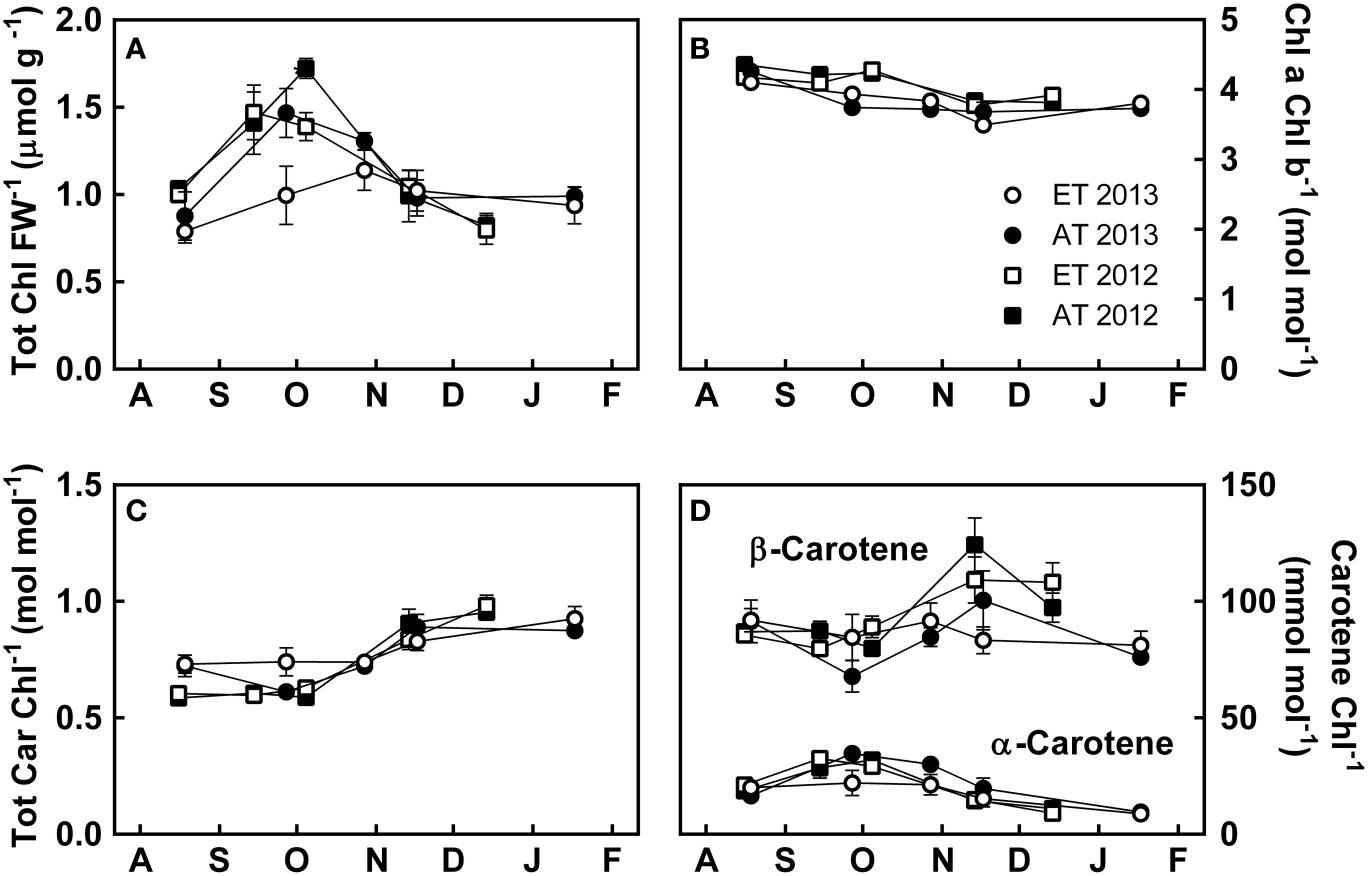
**Effect of elevated temperature on photosynthetic pigments in needles of field-grown white pine seedlings during autumn**. **(A)** Chlorophyll a + b per fresh weight; **(B)** ratio of chlorophyll a to chlorophyll b; **(C)** total carotenoids per total chlorophyll; **(D)** α- and β-carotene per total chlorophyll. AT and ET, seedlings grown at ambient and elevated temperature, respectively. Each data point represents the average of 5 plots, ± S.E.

Total chlorophylls, measured on a fresh-weight basis, increased from August to September, decreased from October to November, and remained stable in December and January (Figure 5A). Chlorophyll a/b decreased, albeit not significantly, from August to December (Figure 5B), whereas total carotenoids increased from October to November and stabilized in December (Figure 5C). β-carotene showed large variations during the autumn and between the treatments (Figure 5D). β-carotene levels increased over October and November, while α-carotene decreased over the same period (Figure 5D). Inter-annual variations were observed in chlorophyll and carotenoid pools, with chlorophyll a/b decreasing earlier in 2013 than in 2012 (Figure 5B), and slightly higher carotenoid levels during August 2013 compared to August 2012 (Figure 5C), though these differences were not significant.

**Figure 6:**
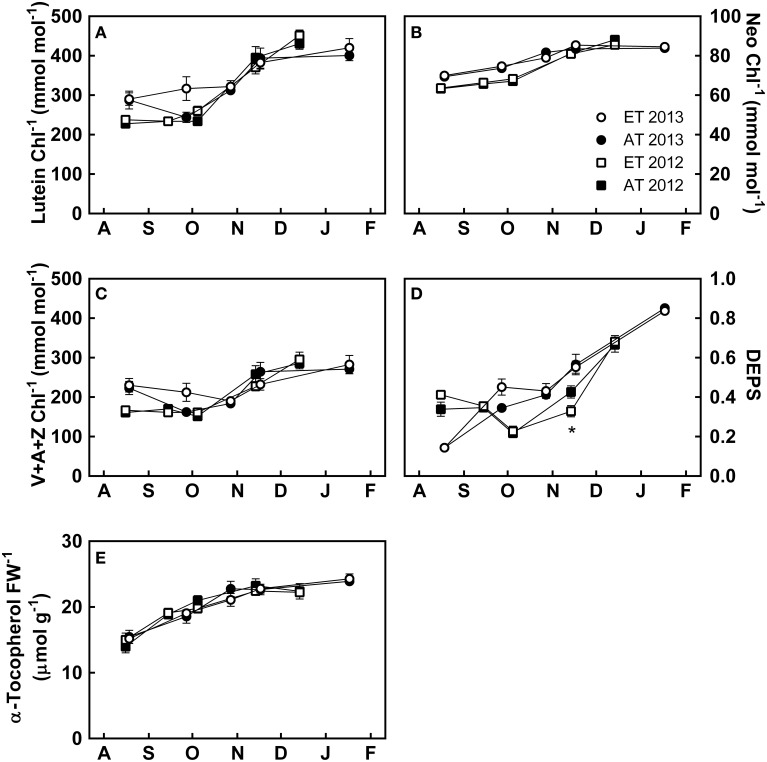
**Effect of elevated temperature on photoprotective metabolites in needles of field-grown white pine seedlings during autumn**. **(A)** Lutein per total chlorophyll; **(B)** neoxanthin per total chlorophyll; **(C)** xanthophyll pool size per total chlorophyll; **(D)** de-epoxidation state (DEPS); **(E)** α-tocopherol per fresh weight. AT and ET, seedlings grown at ambient and elevated temperature, respectively. Each data point represents the average of 5 plots, ± S.E. Asterisks represent significant treatment effect at a single measuring date (^*^P < 0.05).

Photoprotective pigments and metabolites also showed distinct changes during the autumn. Lutein (Figure 6A), neoxanthin (Figure 6B), and xanthophyll cycle pigments (Figure 6C) accumulated through October and November to maximal levels in December. The de-epoxidation status of the xanthophyll-cycle pigments (DEPS) (Figure 6D) transiently increased from October through January. α-tocopherol increased from August to November, followed by stabilization in December (Figure 6E).

Accumulation of lutein (Figure 6A), neoxanthin (Figure 6B), total xanthophylls (Figure 6C) and DEPS (Figure 6D) varied between years. In August 2012, seedlings exhibited low levels of lutein (Figure 6A) and total xanthophylls (Figure 6C) compared to August 2013. DEPS was increased in August 2012 compared to August 2013 (Figure 6D). Increases in lutein (Figure 6A), neoxanthin (Figure 6B), and xanthophylls (Figure 6C) occurred during August and September 2013 which were not observed in 2012. Treatment did not have a significant effect on any of the photosynthetic pigments, whereas time had a significant effect on chlorophyll a/b, α-carotene, β-carotene, DEPS, and neoxanthin (Table 3).

### Control of low temperature and photoperiod on the downregulation of photosynthesis and development of sustained NPQ

**Figure 7:**
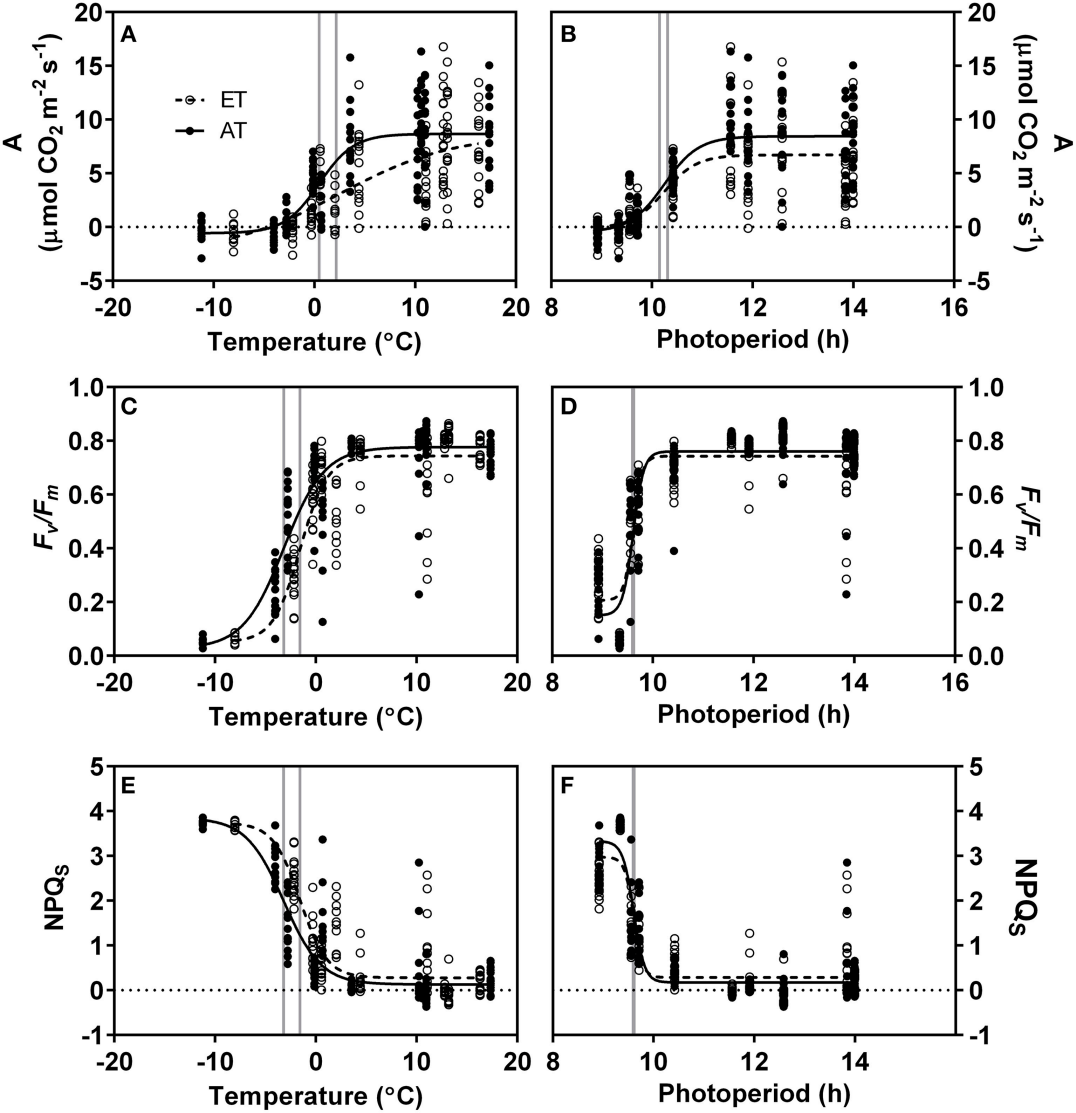
**Relationship of photosynthesis and sustained nonphotochemical quenching with minimum daily temperature and photoperiod**. **(A,B)**, Photosynthetic carbon assimilation **(A)**; **(C,D)**, maximum quantum yield of PSII (F*_v_*/F*_m_*); **(E,F)**, Sustained nonphotochemical quenching (NPQ*_S_*). Each point indicates a single measurement. Lines represent 4-parametric sigmoidal curves fit to the data using the least-squares method. Open circles and dashed lines, elevated temperature (ET); closed circles and solid lines, ambient temperature (AT). Grey lines indicate midpoint of curve at which estimated values represent 50% of the maximum parameter value.

In response to the decrease in daily minimum temperature from 10 to −2°C during autumn and winter, we observed a transient decrease in *A*, *F_v_*/*F_m_* and NPQ*_S_* (Figures 7A,C,E, Table 4). *A* also showed a response to decreasing photoperiod over a range of 11–9 h (Figure 7B, Table 4). In contrast, rapid downregulation of *F_v_*/*F_m_* and rapid induction of sustained NPQ were observed when photoperiod reached a threshold value of approximately 9.6 h (Figures 7D,F, Table 4). Elevated temperature did not significantly affect the response of assimilation to minimum temperature, but did shift the response of *F_v_*/*F_m_* and NPQ*_S_* (Table 4).

**Table 4:** **Curve parameters of 4-parametric sigmoid models presented in Figure 7**.

**Parameter**	**Factor**	**Treat**	**95% CI**	**R^2^**	**Mid (°C)**	**Mid (h)**	***P*_Mid_**	**Slope**	***P*_slope_**
Assimilation	Temperature	AT	1.07	0.63	0.5		0.394	0.25	0.206
		ET	3.24	0.47	2.2			0.11	
	Photoperiod	AT	0.33	0.62		10.3	0.559	1.44	0.820
		ET	0.34	0.46		10.2		1.26	
Fv/Fm	Temperature	AT	0.63	0.82	−3.2		**0.002**	0.23	0.151
		ET	−0.47	0.79	−1.6			0.34	
	Photoperiod	AT	0.05	0.81		9.6	0.418	4.09	0.381
		ET	0.05	0.77		9.6		4.95	
NPQs	Temperature	AT	−0.58	0.82	−3.2		**0.002**	−0.23	0.276
		ET	−0.47	0.79	−1.6			−0.34	
	Photoperiod	AT	0.05	0.83		9.6	0.502	−3.99	0.310
		ET	0.05	0.77		9.6		−4.95	

*Mid indicates the midpoint of the curve at which estimated values represented 50% of the maximum value. For each model curve, mid and slope values were compared between treatments using an extra sum-of-squares F test; bolded values (P < 0.05) indicate statistical significance*.

### Nonstructural carbohydrates

**Figure 8:**
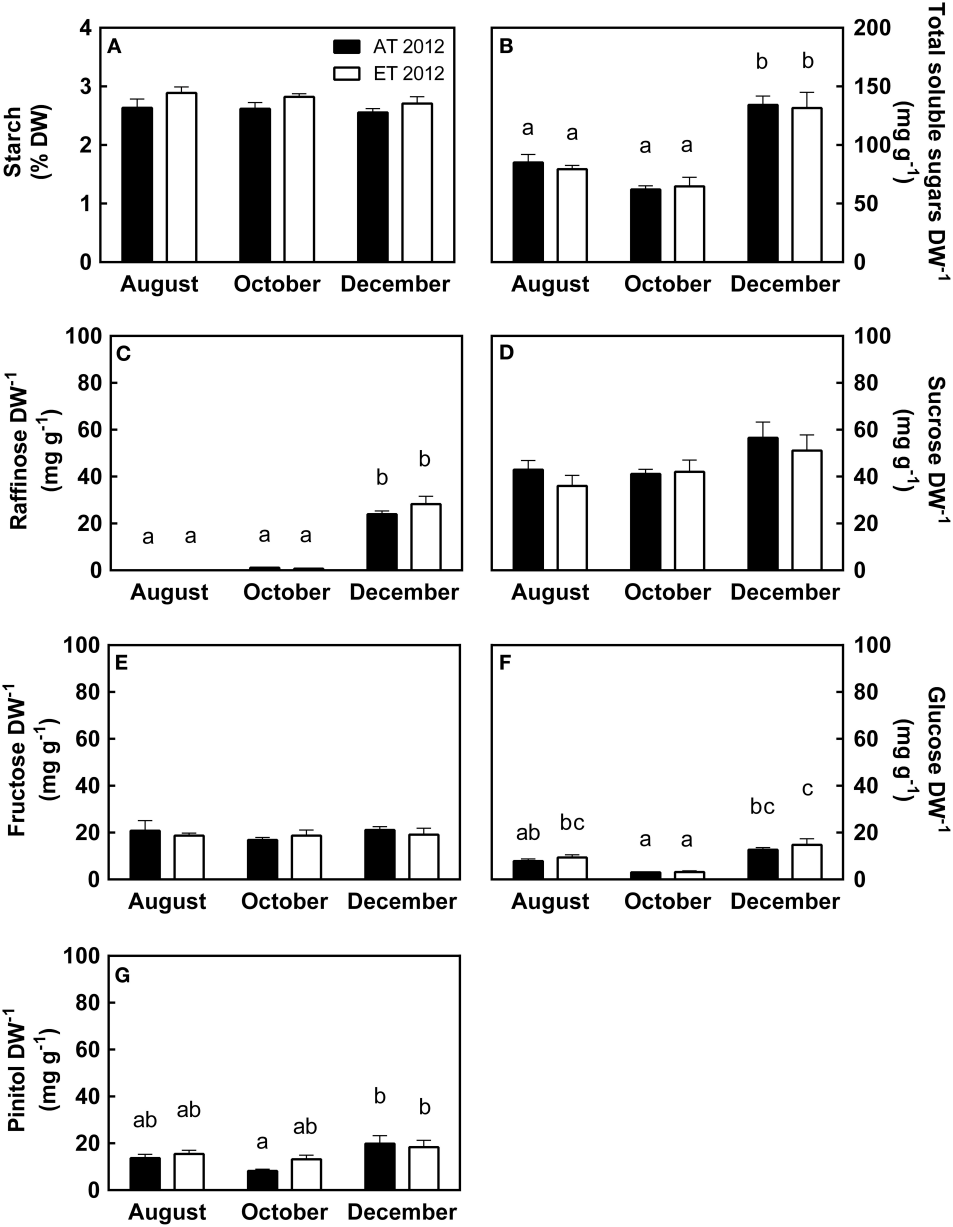
**Effect of elevated temperature on nonstructural carbohydrates in needles of field-grown white pine seedlings during autumn**. **(A)** Leaf starch content, expressed as percent dry weight; **(B)** Total leaf soluble sugars, composed of the sum of **(C–F)**; **(C)** Leaf raffinose content; **(D)** Leaf sucrose content; **(E)** Leaf fructose content; **(F)** Leaf glucose content; **(G)** Leaf pinitol content, expressed per unit dry weight. Samples were collected during August, October and December of 2012. AT and ET, seedlings grown at ambient and elevated temperature, respectively. Each bar represents the average of 5 plots, ± S.E. Letters, where present, indicate significantly different groups (*P* < 0.05).

In both treatments, leaf starch levels remained unchanged from August through December (Figure 8A), while the amount of total soluble sugars decreased slightly from August to October and doubled in December (Figure 8B). Raffinose was absent in August, present in minute quantities in October, and present in large quantities in December (Figure 8C). Sucrose levels were constant during August and October but increased in December (Figure 8D). Fructose levels remained constant from August through December (Figure 8E), while glucose levels decreased by more than 50% from August to October, but tripled from October to December (Figure 8F). Pinitol mirrored the glucose levels, and was reduced by 50% from August to October, but doubled in December (Figure 8G). Seedlings in the heated plots exhibited significantly higher amounts of leaf starch (*P* = 0.026, Table S1); however, this did not significantly affect the accumulation of soluble sugars (Table S1).

### Freezing tolerance

**Figure 9:**
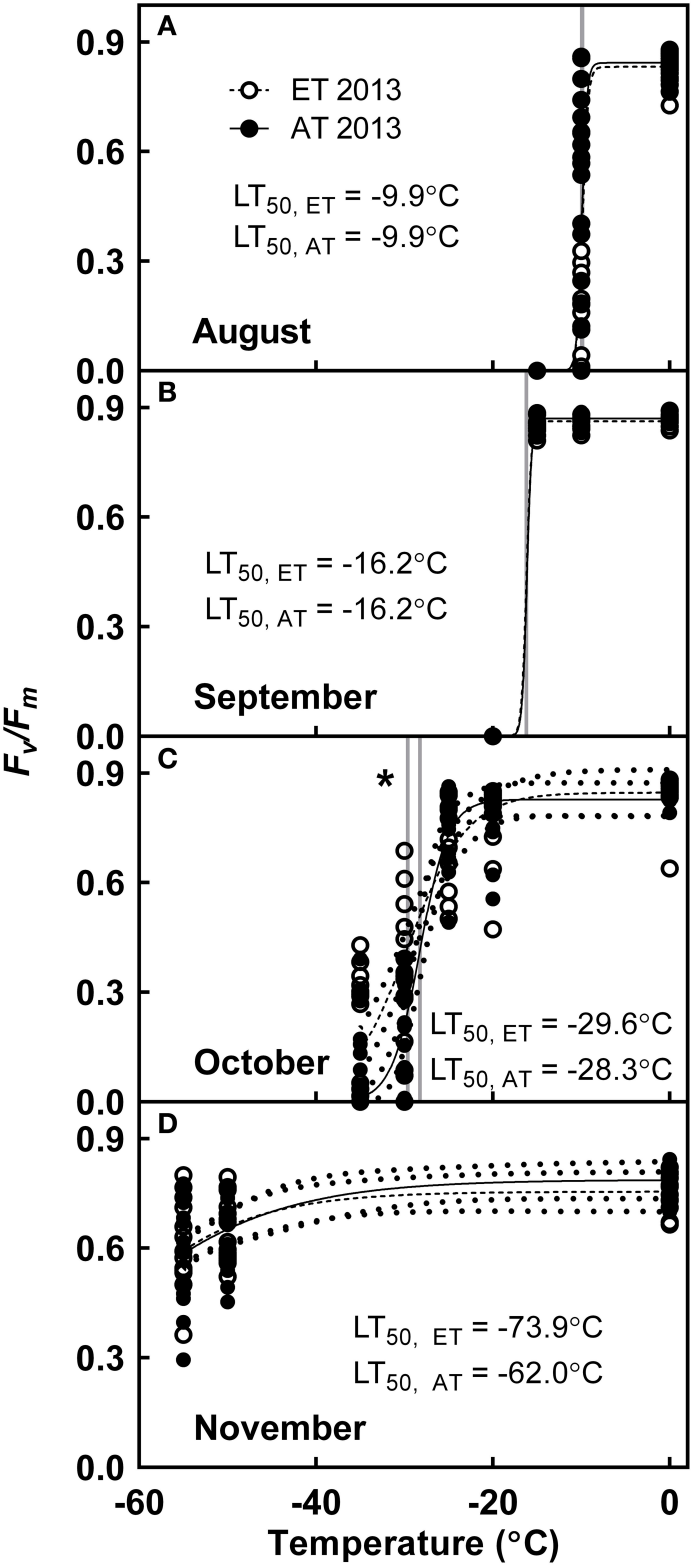
**Effect of elevated temperature on cold hardening in field-grown white pine seedlings during autumn**. Freezing tolerance was assessed in **(A)** August; **(B)** September; **(C)** October; and **(D)** November of 2013. Dotted lines, where present, indicate 95% confidence interval (CI) of model. AT and ET, seedlings grown at ambient and elevated temperature, respectively. Solid line indicates ambient temperature model; dashed line indicates elevated temperature model. Gray lines indicate LT_50_. Asterisk indicates significant treatment effect at LT_50_ (^*^P < 0.05).

In 2013, seedlings exhibited tolerance to freezing exposure of −10°C during August (Figure 9A), −16°C during September (Figure 9B), −30°C in October (Figure 9C) and were fully cold hardy below −60°C by November (Figure 9D). The cold hardiness of ET seedlings from heated plots did not differ from that of Control seedlings from unheated plots during August (Figure 9A) or September (Figure 9B). In October, seedlings from heated plots exhibited significantly greater freezing tolerance in comparison with freezing tolerance of seedlings from unheated plots (*P* = 0.027, Figure 9C).

## Discussion

We explored the effect of a moderate increase of air temperature by +1.5°C during the day and +3°C during the night on the development of cold acclimation by assessing photosynthesis, photoprotective NPQ and pigments, carbohydrate metabolism and freezing tolerance in Eastern white pine in a field experiment. We observed that physiological responses of ET seedlings exposed from heated plots mainly differed from unheated Control seedlings during August, September and early October. We also observed that most physiological changes in photosynthesis (Figures 2, 3) and cold hardiness (Figure 9) occurred during late September and early October, while photoprotective modifications of energy quenching characteristics and pigment composition (Figures 3, 6) occurred later, during November and December. Our data clearly suggests that under field conditions, an increase in temperature by 1.5°C during the day and 3°C during the night does not extend the length of the growing season and does not delay the downregulation of photosynthesis, the increase in photoprotective capacity, accumulation of nonstructural carbohydrates, or development of freezing tolerance in *Pinus strobus* seedlings.

### Photosynthesis

#### Elevated temperature affects photosynthetic gas exchange during the growing season

During August and September of both years, photosynthetic gas exchange in ET seedlings was significantly different compared to Control seedlings (Table 3). Throughout our measurements, stomatal conductance was below 0.15 mol H_2_O m^−2^ s^−1^, the threshold at which stomatal conductance begins to limit Rubisco activity (Flexas et al., 2004). In seedlings growing under elevated temperature conditions, stomatal conductance was decreased (Figure 2B) and contributed to reduced assimilation (Figure 2A) and evapotranspiration (Figure 2D). ET seedlings from the heated plots exhibited improved water use efficiency from August through October in 2012, but this effect was not observed in 2013 (Figure 2C). During August, stomatal conductance of both treatments was higher in 2013 than in 2012 (Figure 2B), though assimilation for both treatments was greatly reduced in 2013 (Figure 2A), suggesting that stomatal conductance did not limit assimilation in August 2013. As neither PSII activity (Figure 3B) nor excitation pressure (Figure 3D) differed between August 2012 and 2013, the limiting factor of assimilation was likely a decreased sink capacity of the seedlings. Measurements in September 2013 were taken after nighttime temperature fell below 5°C, and resulted in decreased stomatal conductance although assimilation remained high; again, neither PSII activity nor excitation pressure were affected.

#### Elevated temperature decreases stomatal conductance and evapotranspiration even in the absence of water stress

Seedlings exposed to elevated temperature consistently exhibited lower stomatal conductance (Figure 2B) and evapotranspiration (Figure 2D) during the growing season. In 2013, we assessed soil moisture and water potential in order to determine whether gas exchange during the growing season was responding to water stress imposed by the infrared heating method. We did not observe a significant decrease in soil moisture in elevated temperature plots (Figure 4A). Furthermore, pre-dawn and midday water potential measurements from both Control and ET seedlings were greater than −0.2 MPa (Figures 4B,C). Since osmotic stress is typically incurred when water potential falls below −1.0 MPa (Flexas et al., 2004; Verslues et al., 2006), we concluded that the decrease in gas exchange observed in the elevated temperature treatment was not a result of osmotic stress.

We also assessed air temperature and humidity during July-August of 2014 and recorded a 6% increase in daytime VPD in heated plots (Figure S1). A recent study which modeled water loss in response to infrared heating predicted a 12–15% increase in transpiration, but noted that certain species such as *Populus tremuloides* would exhibit reduced transpiration as a result of reduced stomatal conductance (de Boeck et al., 2012). Therefore, enhanced VPD may have contributed to the decrease in stomatal conductance observed in our elevated temperature treatment during the growing season. We conclude that when subjected to elevated temperature, *P*. *strobus* pre-emptively reduces stomatal conductance in an attempt to prevent excessive water loss via evapotranspiration at the cost of reduced photosynthesis.

#### Downregulation of photosynthetic gas exchange is driven by both temperature and photoperiod, and is not delayed in seedlings from heated plots

Downregulation of photosynthetic gas exchange between October and November was strongly correlated with air temperature (Table 4) and commenced once nighttime temperatures decreased below 10°C, irrespective of treatment (Figures 2A, 7A). The transient downregulation of photosynthesis occurred with the decrease in temperature at a rate of 0.25 μmol CO_2_ m^−2^ s^−1^/°C in Control seedlings, and at a rate of 0.11 μmol CO_2_ m^−2^ s^−1^/°C in ET seedlings (Table 4). However, the reason for the lower rate of the downregulation of photosynthesis in ET seedlings largely reflects the fact that photosynthesis in seedlings from the heated plots was already decreased during the growing season compared to seedlings from unheated plots. No photosynthetic gas exchange was observed when temperatures decreased below −2°C, irrespective of treatment (Figure 7A), but this complete downregulation occurred earlier in 2012 than in 2013 (Figure 2), following the earlier occurrence of night frosts in 2012 (Figure 1B). The effect of air temperature on seasonal variations in photosynthesis was previously modeled by Bergh et al. (1998) for *Picea abies* and Mäkelä et al. (2004) for *Pinus sylvestris*. Downregulation of photosynthesis following a decrease in air temperature below 0°C was also observed in *P. sylvestris* stands in northern Sweden (Strand et al., 2002) and Siberia, Russia (Lloyd et al., 2002). In addition to air temperature, our data also reveal that photosynthetic gas exchange decreased by 50% as photoperiod decreased to 10 h, irrespective of treatment, and was completely absent at a 9 h photoperiod (Figure 7B). The transient response of carbon assimilation to both temperature and photoperiod signals indicates that gas exchange is modulated in concert with decreased metabolic activity (Rossi et al., 2008) and leaf carbon export (Hoch et al., 2003). We conclude that both low temperature and photoperiod exerted a strong control on the downregulation of photosynthetic gas exchange.

#### Downregulation of light reactions is preceded by downregulation of gas exchange and is not affected by elevated temperature

*F_v_*/*F_m_* began to decrease after photoperiod had decreased below 10 h and after the occurrence of nighttime frosts (Figure 1B). Despite the significant effect of the elevated temperature treatment on gas exchange in ET seedlings during the downregulation of photosynthetic CO_2_ uptake between October and November, *F_v_*/*F_m_* and Φ_PSII_ in seedlings from the heated plots were similar to values observed in seedlings from unheated control plots (Table 3). Further downregulation of *F_v_*/*F_m_* occurred throughout December and January, while photosynthetic gas exchange had already ceased by December (Figures 2A, 3B). The transient seasonal changes observed for *F_v_*/*F_m_* and Φ_PSII_ result from the reorganization of thylakoid membrane-bound photosynthetic proteins. This has been demonstrated e.g. for D1 and LHCII protein content in needles of *P. strobus* (Verhoeven et al., 2009) and *P. sylvestris* (Ottander et al., 1995; Ensminger et al., 2004). These adjustments occur even when temperatures are consistently below 0°C. Given the sequence of the observed events, it therefore appears that the downregulation of photosynthetic gas exchange and hence Calvin cycle activity precedes reorganization of the photosynthetic apparatus in the thylakoid membrane during autumn.

Least squares curve fitting (Table 4) revealed that variation in *F_v_*/*F_m_* was strongly correlated with both photoperiod and temperature. However, while decreases in temperature during autumn resulted in a transient decrease of *F_v_*/*F_m_* (Figure 7C), we observed an instant response of *F_v_*/*F_m_* to photoperiod when daylength decreased to 9.6 h (Figure 7D). These observations indicate that the reorganization of the photosynthetic apparatus and the downregulation of the light reactions are more sensitive to photoperiod than temperature, but also that photoperiodic regulation of the light reactions operates on a threshold rather than a gradient basis.

### Photoprotective nonphotochemical quenching and pigment dynamics

#### Sustained nonphotochemical quenching develops after the downregulation of photosynthetic gas exchange and is not delayed by elevated temperature

From August to November, excess light energy was efficiently quenched by dynamic xanthophyll cycle-mediated NPQ (Figure 3C). The transition from dynamic NPQ to winter sustained NPQ occurred synchronously with the downregulation of *F_v_*/*F_m_* from November through January (Figure 3C), as photoperiod decreased below 10 h and nighttime temperatures decreased below 0°C (Figures 1B, 7). The development of sustained NPQ in response to low temperature (Figure 3C) is correlated with the retention of antheraxanthin and zeaxanthin (Adams and Demmig-Adams, 1994; Savitch et al., 2002) and results in increased DEPS (Figure 6D). In contrast to our expectations, the transition from dynamic to sustained NPQ was not significantly delayed in seedlings from the heated plots, since the sustained quenching occurred in parallel in both Control and ET seedlings (Figure 3C).

#### Elevated temperature does not affect pigment pool size

Photosynthetic pigments in ET seedlings from the heated plots did not reveal any significant differences when compared to Control seedlings from unheated plots, indicating that moderately elevated temperature did not impact the pool sizes of chlorophylls or carotenoid pigments (Figure 5, Table 3). Nonetheless, we observed major changes in pigment composition during autumn in *Pinus strobus* seedlings, which are consistent with development of sustained nonphotochemical quenching. Total chlorophylls (Figure 5A) and α-carotene levels (Figure 5D) increased from August to early October, following the transient increase in assimilation (Figure 2A), and decreased again during November when assimilation decreased (Figures 2A, 3B). While photosynthesis and total chlorophyll levels decreased from October onwards, the pool of total carotenoids showed the opposite trend and nearly doubled from October to November (Figure 5C). This was mainly due to the increase of the photoprotective lutein and xanthophyll cycle pigments (Figures 6A,C). Increases in these pigments during autumn have been previously observed in pine species as well as *Pseudotsuga menziesii* and *Picea pungens* (Adams and Demmig-Adams, 1994; Ensminger et al., 2004; Verhoeven et al., 2009) and contribute to alleviate the enhanced risk of photo-oxidative damage (Ensminger et al., 2004).

β-carotene, a component of both reaction centers and core antenna, serves dual functions as an accessory pigment (Trebst, 2003) and also as a biosynthetic precursor to zeaxanthin (Bartley and Scolnik, 1995). We observed transient accumulation of β-carotene from October to November followed by a decrease in December, concurring with results reported by Verhoeven et al. (2009). Our results suggest that the β-carotene accumulated during this period is converted to zeaxanthin during the development of winter sustained nonphotochemical quenching, as β-carotene (Figure 5D) and NPQ (Figure 3C) responded similarly during late autumn. The accumulation of pigments involved in photoprotective quenching of excess light (Figures 6A–C,E) was completed by November and thereby also indicated the complete cessation of photosynthetic gas exchange (Figures 2A, 3B).

### Non-structural carbohydrates

#### Elevated temperature increases leaf starch content during autumn

Starch levels in mature needles were low (2.5–3% of leaf dry weight) during August, October and December of 2012 (Figure 8A), consistent with autumn starch levels reported previously (Little, 1970; Pomeroy et al., 1970; Hoch et al., 2003). Elevated autumn temperature caused a small but significant increase in needle starch content, indicated by higher starch levels in ET seedlings from the heated plots during the period October to December 2012 (Table S1). The reason for this increase is unclear. Typically growth at elevated temperature results in depletion of starch due to the associated increases in foliar respiration (Geigenberger, 2011). However, we did not observe an increase in respiration in seedlings from heated plots. In addition, the increase in starch cannot be explained by increased assimilation, since rates of assimilation were always lower in ET seedlings than in Control seedlings in 2012. However, increases in leaf starch content resulting from elevated temperature have been reported recently by Glaubitz et al. (2014). They observed accumulation of leaf carbohydrates including starch in some *Oryza* cultivars in response to asynchronous elevated night time temperature. In another study, Zhao et al. (2012) showed an increase in leaf starch content in poplar leaves when growing under elevated temperature. At this point, the cause for the increased starch levels in seedlings from heated plots remains unclear and deserves further investigation.

#### Accumulation of soluble carbohydrates during autumn occurs in response to low temperature in needles of ET and control seedlings

There was no significant difference in soluble carbohydrate content of needles from heated or control plots (Figure 8; Table S1), indicating that soluble carbohydrate metabolism did not respond to the elevated temperature treatment. However, cold acclimation during the autumn was associated with major changes in the carbohydrates assessed in our study. In August, total soluble sugars were mainly comprised of fructose and sucrose (Figure 8). During August, we also observed the presence of moderate amounts of pinitol (2% of leaf dry weight), an osmoprotectant with cryoprotective characteristics (Angelcheva et al., 2014); these levels concur with levels observed during the growing season in *P. sylvestris* (Ericsson, 1979). The majority of changes in soluble sugar levels occurred between October and December. When nighttime temperatures decreased to below 10°C (Figure 1B), seedlings began to adjust to low temperature and short photoperiod. Aside from raffinose, which increased by over 20-fold from October to December (Figure 8C), increases in carbohydrate levels remained between the 1–2 fold range (Figures 8D–G), as expected for glucose and pinitol (*P. sitchensis*, Dauwe et al., 2012; *Picea obovata*, Angelcheva et al., 2014). Raffinose is known to accumulate significantly in response to low temperature (Strimbeck et al., 2008; Dauwe et al., 2012; Angelcheva et al., 2014). Raffinose is also associated with the enhancement of freezing tolerance (Strimbeck et al., 2008), and has been shown to increase PSII stability during freeze-thaw cycles in *Arabidopsis thaliana* (Knaupp et al., 2011).

We observed a 30% increase in sucrose from October to December (Figure 7D), which concurs with previously reported levels in other conifer species (Strimbeck et al., 2008; Dauwe et al., 2012) but is much lower than the 5-fold increase previously reported for *P. strobus* (Hinesley et al., 1992). The rather small changes in leaf soluble carbohydrate content observed here may be a consequence of the mild winter in 2012 (Figure 1B). Even so, by December, the amount of total soluble carbohydrates had doubled (Figure 8B), of which 25% were represented by raffinose, which was absent in samples from August and October (Figure 8C). This shift in leaf carbohydrate composition likely improved winter freezing tolerance.

### Cold hardiness

#### Freezing tolerance is first induced by photoperiod, and is not impaired by elevated autumn temperature

ET seedlings from the heated plots did not exhibit delayed induction of cold hardening in August and September, and in contrast to our hypothesis, freezing tolerance was not impaired in seedlings from the heated plots. Growth at moderately elevated temperature instead appeared to enhance freezing tolerance in ET seedlings in October and November (Figure 9C). This concurs with a previous study on *P. sylvestris*, which revealed that there was no effect of elevated temperature on the induction of cold hardening and freezing tolerance during midwinter (Repo et al., 1996).

In 2013, seedlings were already tolerant to freezing exposure at −10°C in August (Figure 9A), and their freezing tolerance continued to increase in September to −16°C (Figure 9B), which is within the ranges previously reported for freezing tolerance in summer-acclimated conifers (Strimbeck et al., 2008). Four days after the first frost in October (Figure 1B), freezing tolerance further increased to −30°C (Figure 9C), and a significant treatment effect (*P* < 0.05) was observed with enhanced freezing tolerance exhibited by the elevated temperature treatment. By November, following several weeks of exposure to night temperatures below 0°C (Figure 1B), seedlings of both treatments were fully cold hardened, with freezing tolerance below −60°C (Figure 9). We conclude that the initial stages of cold hardening during early autumn are triggered by decreasing photoperiod; similar observations have previously been reported in *Populus tremula* × *tremuloides* (Welling et al., 2002), *Betula pubescens* (Welling et al., 2004) and *Picea abies* (Rostad et al., 2006). The addition of the low temperature signal in October greatly increased the development of freezing tolerance.

## Conclusions

In contrast to our initial hypotheses, we did not observe a significant delay in the downregulation of photosynthesis or cold hardening when seedlings were exposed to elevated temperature in heated plots, nor did these seedlings exhibit altered carbohydrate metabolism or impaired cold hardiness. Though exposure to +1.5/+3°C in heated plots was insufficient to delay autumn cold acclimation, it was sufficient to decrease photosynthesis during the growing season and enhance nonphotochemical quenching. Our data further indicate that the downregulation of photosynthetic gas exchange occurs synchronously with the accumulation of photoprotective carotenoids, accumulation of soluble sugars and early stages of cold hardening, but its timing precedes the downregulation of the light reactions and the transition from dynamic NPQ to sustained NPQ. We also observed that the autumn physiology of *P. strobus* is most sensitive to elevated temperature during the transition starting at the beginning of the downregulation of photosynthesis and during the development of cold hardiness.

Based on our findings it seems unlikely that *P. strobus* seedlings will be significantly affected by the moderately elevated autumn temperatures used in our experiment. However, we have shown that the sensitivity of *P. strobus* seedlings to elevated temperature is increased under water-limited or chilling conditions. We have further demonstrated that a +1.5/+3°C increase in elevated temperature will not significantly extend the growing season or adversely affect cold acclimation. Instead it appears that moderate increases in elevated temperature will affect productivity during the growing season, when *P. strobus* may compromise photosynthetic CO_2_ uptake under water-limiting conditions, whereas elevated temperature during autumn does not necessarily increase the carbon uptake period and extend the growing season length in this evergreen conifer.

## Author contributions

CC and IE designed the study. CC performed field measurements and samplings. CC and AZ developed the freezing test protocol. AZ performed the freezing tests with input from CC. FU and SM developed the carbohydrate HPLC protocol. FU performed carbohydrate analyses with input from SM. CC performed pigment and data analyses. CC and IE wrote the manuscript. All authors read and approved the final manuscript.

### Conflict of interest statement

The authors declare that the research was conducted in the absence of any commercial or financial relationships that could be construed as a potential conflict of interest.
