# Supplementary material for: Sensitivity of cold acclimation to elevated autumn temperature in field-grown Pinus strobus seedlings
Source: Front Plant Sci. 2015 Mar 24;6:165. doi: 10.3389/fpls.2015.00165 (PMC4371696; doi:10.3389/fpls.2015.00165)
Supplement: Supplementary file 1 [file Table1.DOCX]

Supplementary Material

Sensitivity of cold acclimation to elevated autumn temperature in field-grown *Pinus strobus* seedlings

**Christine Y. Chang^1,3^, Faride Unda^2^, Alexandra Zubilewich^1^, Shawn D. Mansfield^2^, Ingo Ensminger^1,3,4*^**

^1^ Department of Biology, University of Toronto Mississauga, Mississauga, ON, Canada

^2^ Department of Wood Science, University of British Columbia, Vancouver, BC, Canada

^3^ Graduate Department of Cell & Systems Biology, University of Toronto, Toronto, ON, Canada

^4^ Graduate Department of Ecology and Evolutionary Biology, University of Toronto, Toronto, ON, Canada

*** Correspondence:** Ingo Ensminger, Department of Biology, University of Toronto at Mississauga, 3359 Mississauga Road N., Mississauga ON, L5L1C6, Canada.

[ingo.ensminger@utoronto.ca](mailto:ingo.ensminger@utoronto.ca)

# Supplementary Tables

Table S1. Summary of two-way ANOVA analysis showing the effects of time, treatment and their interaction on leaf carbohydrate concentrations. *P-*values in bold indicate statistical significance (α = 0.05).

|  | **Treatment** | | **Time** | | **Treatment x Time** | |
| --- | --- | --- | --- | --- | --- | --- |
| **Variable** | ***F*** | ***P*** | ***F*** | ***P*** | ***F*** | ***P*** |
| Starch | 5.641 | **0.026** | 0.799 | 0.461 | 0.112 | 0.894 |
| Raffinose | 1.193 | 0.286 | 202.100 | **<0.001** | 1.534 | 0.236 |
| Sucrose | 0.843 | 0.368 | 4.605 | **0.020** | 0.330 | 0.722 |
| Glucose | 1.401 | 0.248 | 33.670 | **< 0.001** | 0.276 | 0.761 |
| Fructose | 0.166 | 0.687 | 0.411 | 0.668 | 0.352 | 0.707 |
| Pinitol | 0.903 | 0.351 | 7.266 | **0.003** | 1.067 | 0.360 |
| Total soluble sugars | <0.001 | 0.993 | 21.440 | **< 0.001** | 0.358 | 0.703 |

Figure S1. Effects of infrared heating on air temperature and vapor pressure deficit (VPD). A) Temperature increment in heated plots, B) VPD increment in heated plots, and C) daily precipitation measured during July 17-August 26, 2014 at Koffler Scientific Reserve, King City, Ontario.
